# Correlations between multimodal neuroimaging and peripheral inflammation in different subtypes and mood states of bipolar disorder: a systematic review

**DOI:** 10.1186/s40345-024-00327-w

**Published:** 2024-02-22

**Authors:** Jing-Yi Long, Bo Li, Pei Ding, Hao Mei, Yi Li

**Affiliations:** 1 Wuhan Mental Health Center, No. 89, Gongnongbing Rd., Jiang’an District, Wuhan, 430012 Hubei Province China; 2 Affiliated Wuhan Mental Health Center, Tongji Medical College of Huazhong University of Science and Technology, Wuhan, China; 3 School of Public Administration, China University of Geosciences, Wuhan, 430074 China; 4 Zhongnan Hospital of Wuhan University, No. 169, East Lake Rd., Wuchang District, Wuhan, 430062 Hubei Province China

**Keywords:** Bipolar disorder, Peripheral inflammation, Neuroimaging, Brain, Fronto-limbic-striatal system

## Abstract

**Background:**

Systemic inflammation-immune dysregulation and brain abnormalities are believed to contribute to the pathogenesis of bipolar disorder (BD). However, the connections between peripheral inflammation and the brain, especially the interactions between different BD subtypes and episodes, remain to be elucidated. Therefore, we conducted the present study to provide a comprehensive understanding of the complex association between peripheral inflammation and neuroimaging findings in patients with bipolar spectrum disorders.

**Methods:**

This systematic review was registered in the International Prospective Register of Systematic Reviews (PROSPERO) database (CRD42023447044) and conducted according to the Population, Intervention, Comparison, Outcomes, and Study Design (PICOS) framework. Online literature databases (PubMed, Web of Science, Scopus, EMBASE, MEDLINE, PsycINFO, and the Cochrane Library) were searched for studies that simultaneously investigated both peripheral inflammation-related factors and magnetic resonance neurography of BD patients up to July 01, 2023. Then, we analysed the correlations between peripheral inflammation and neuroimaging, as well as the variation trends and the shared and specific patterns of these correlations according to different clinical dimensions.

**Results:**

In total, 34 publications ultimately met the inclusion criteria for this systematic review, with 2993 subjects included. Among all patterns of interaction between peripheral inflammation and neuroimaging, the most common pattern was a positive relationship between elevated inflammation levels and decreased neuroimaging measurements. The brain regions most susceptible to inflammatory activation were the anterior cingulate cortex, amygdala, prefrontal cortex, striatum, hippocampus, orbitofrontal cortex, parahippocampal gyrus, postcentral gyrus, and posterior cingulate cortex.

**Limitations:**

The small sample size, insufficiently explicit categorization of BD subtypes and episodes, and heterogeneity of the research methods limited further implementation of quantitative data synthesis.

**Conclusions:**

Disturbed interactions between peripheral inflammation and the brain play a critical role in BD, and these interactions exhibit certain commonalities and differences across various clinical dimensions of BD. Our study further confirmed that the fronto-limbic-striatal system may be the central neural substrate in BD patients.

**Supplementary Information:**

The online version contains supplementary material available at 10.1186/s40345-024-00327-w.

## Introduction

Substantial evidence has demonstrated that a dysregulated inflammatory/immune state has wide-ranging and far-reaching impacts on bipolar disorder (BD) (Sewell et al. 2021; Modabbernia et al. 2013; Brietzke et al. 2009; Kunz et al. 1999). In the early stages of BD, patients often suffer from upregulation of inflammatory pathways, which is primarily characterized by elevated levels of peripheral inflammation. Moreover, chronic inflammatory activation is recognized as an essential component of the key mechanisms of BD pathogenesis. For instance, individuals at risk for BD with higher baseline levels of interleukin (IL)-6, tumour necrosis factor (TNF)-α, and C-reactive protein (CRP) and adolescents with asthma were more prone to developing BD (Chen et al. 2014, 2020b). Notably, dysregulated immune-inflammatory signalling affects BD patients of almost all ages (Karthikeyan et al. 2022; Tsai et al. 2019), and autopsy evidence has indicated increased levels of peripheral inflammation in postmortem brain samples from BD patients (Giridharan et al. 2020). Moreover, the impacts of peripheral inflammatory activation on patients with BD typically involve multiple systems or organs that are crucial for the progression of this disease. Therefore, BD is increasingly considered a multisystem inflammatory disease (Leboyer et al. 2012).

Numerous multimodal magnetic resonance imaging (MRI) findings have consistently emphasized that brain alterations in BD patients are diverse and involve functional activity and functional connectivity, morphology (e.g., volume, cortical thickness), white matter microstructure, and metabolism. Magnetic resonance spectroscopy (MRS) studies have confirmed that dysfunctions in the glutamatergic system participate in the pathophysiological process of BD (Zarate et al. 2005; Benedetti et al. 2009). Morphological clues revealed a progressive decline in grey matter and white matter volume from early to late developmental stages, which may be neurodevelopmental deficits that are specific to BD (Chakrabarty et al. 2021). Moreover, the altered areas are diverse and can affect multiple regions throughout the whole brain (Wise et al. 2017); commonly susceptible regions in BD patients include the orbitofrontal cortex (Nery et al. 2009), dorsomedial PFC (Poletti et al. 2019), dorsolateral PFC (Poletti et al. 2017), ventromedial PFC (Savitz et al. 2013), and ventrolateral PFC (Tseng et al. 2021). In addition, changes in the brain are closely related to the patient's behavioural manifestations. For instance, the emotion regulation network, which consists mainly of the prefrontal cortex, amygdala, and cingulate gyrus, shows aberrant activation in patients with BD, and the different activation patterns correspond with distinct affective symptoms (Njau et al. 2020). Thus, as research advances, BD is gradually being regarded as a systemic brain disease (Zhou et al. 2017).

The effects of peripheral inflammation on the brain, and vice versa, might be widespread and profound. The interactions between inflammation and the brain are also related to the clinical characteristics and treatment outcomes of patients with BD. Elevated peripheral levels of high-sensitivity CRP (hs-CRP), for example, have been associated with reduced functional connectivity and grey matter volume in the dorsal caudal putamen and ventrolateral PFC, as well as decreased cognitive function, in patients with BD (Tseng et al. 2021). In contrast, following treatment with TNF-α antagonists, whole-brain cortical thickness increases, and affective symptoms improve in BD patients as serum TNF-α levels decrease (Mansur et al. 2020). Moreover, the hippocampus has been shown to be the region most prone to neuronal loss in patients with BD (Tsai et al. 2019; Inal-Emiroglu et al. 2015), and abnormalities observed in multidimensional neuroimaging are thought to be related to the abnormal activation of microglia as a result of neuroinflammation. Although considerable evidence has demonstrated that inflammatory activation is closely associated with brain alterations in BD (Capuron and Miller 2011), most existing studies have explored a single discipline related to neuroimaging or inflammation; in particular, explorations of the correlations between multimodal neuroimaging and inflammation-related factors based on different BD types and mood states are lacking. Hence, the complicated links between peripheral inflammation and the brain, especially among different clinical dimensions, have yet to be further revealed.

In summary, we conducted the present systematic review to reveal the complex associations between peripheral inflammation and the brain in patients with bipolar spectrum disorders. First, previous studies of BD patients, in which both peripheral inflammation-related factor measurements and magnetic resonance neuroimaging scanning were simultaneously performed, were reviewed. Subsequently, the characteristics of the correlations between peripheral inflammation and neuroimaging were extracted, and the trends, heterogeneity, and consistency were summarized. Furthermore, the shared and specific interaction patterns between peripheral inflammation and the brain in different clinical dimensions of BD were categorized according to different BD subtypes and mood states.

## Methods

### Search strategy and study selection

This systematic review was registered in the International Prospective Register of Systematic Reviews (PROSPERO) database (CRD42023447044) and conducted according to the Preferred Reporting Items for Systematic Reviews and Meta-analyses (PRISMA) checklist (Page et al. 2021). Studies were identified by searching electronic databases, including PubMed, Web of Science, Scopus, EMBASE, MEDLINE, PsycINFO, and the Cochrane Library, from the inception of each database up to July 01, 2023 for studies published in English. The search terms used were (bipolar disorder OR bipolar depression OR bipolar disease OR cyclothymic disorder OR BD) AND (magnetic resonance imaging OR MRI) OR (functional MRI OR fMRI) OR (structural MRI OR sMRI) OR (diffusion tensor imaging OR DTI) OR [magnetic resonance spectroscopy OR MRS) OR (OR ASL)] AND [(inflammation OR inflammatory) OR (immunity OR immune) OR cytokines OR chemokines OR interleukins OR neurotrophic factor] (additional details can be found in Additional file 1). Furthermore, two additional publications were identified through the manual search of reference lists.

### Inclusion and exclusion criteria

In accordance with the PICOS strategy (Participants, Interventions, Comparisons, Outcomes, and Study Design), studies that met the following criteria were included: (1) Participants: males and females of any age with a diagnosis of BD of any subtype and mood state as assessed by the Diagnostic and Statistical Manual of Mental Disorders (DSM) or International Classification Diseases (ICD); (2) Intervention: observational studies in which almost all BD individuals had received or were undergoing some form of therapy, e.g., pharmacological, physical, psychological, or behavioural; (3) Comparison: (a) comparison of BD patients and controls or (b) comparison of different subtypes and mood states in BD patients; (4) Outcomes: studies that collected data on both neuroimaging and peripheral inflammation in patients with BD; all the results included (a) levels of inflammation-related markers (including proinflammatory, anti-inflammatory, chemokine, and neurotrophic factor markers) and (b) alterations in multimodal neuroimaging (including brain function, morphology, structure, and metabolism); and (5) study design: studies that simultaneously explored peripheral inflammation and neuroimaging in BD individuals, including observational studies (cross-sectional, retrospective, prospective, and cohort studies), randomized controlled trials (RCTs), and case series studies. Subsequently, we extracted the alterations in inflammation levels and neuroimaging and analysed the correlations between the two variables. The exclusion criteria were as follows: (1) reviews, meta-analyses, conference abstracts, case reports, theoretical papers, or research protocols; (2) studies with subjects who did not meet the diagnostic criteria for BD; (3) studies in which only peripheral inflammatory or neuroimaging research was conducted; (4) studies with no distinction between patients with BD and patients with other diseases; (5) studies that measured blood markers that were not related to inflammation; and (6) studies with non-brain MRI data (e.g., electroencephalography, abdominal MRI, magnetoencephalography).

### Study selection and data extraction

Two researchers (H.M., B.L.) independently appraised and screened the eligibility of the studies according to the selection criteria, evaluated the full texts of the relevant records and compiled an initial draft of the data summary. Any discrepancies were discussed until a consensus was reached by involving a third senior reviewer (Y.L.). Two investigators (J.Y.L., P.D.) validated the data and extracted the following variables from each article: (1) authors; (2) year of publication; (3) sample size; (4) male-to-female ratio; (5) age; (6) BD subtype; (7) mood state; (8) treatment details; (9) condition of comorbidities; (10) peripheral inflammation-related factors; (11) MRI modality; (12) altered brain regions; (13) neuroimaging relevant indicators, e.g., volume, thickness, functional activity, functional connectivity, white matter integrity, and metabolic levels; and (14) correlations between peripheral inflammation and neuroimaging. Variables that were not described in the articles and for which no information was received from the corresponding author were labelled not applicable (NA). The study was excluded if crucial information was missing or if we did not receive a response from the corresponding author.

### Quality assessment

We followed the Strengthening the Reporting of Observational Studies in Epidemiology (STROBE) (Elm et al. 2007) statement to assess the quality and index of bias of the included studies. The STROBE statement is a widely used risk of bias evaluation tool that focuses on the clinical characteristics of participants to assess the impact of diagnostic heterogeneity, medications, and comorbidities on outcomes. We also included items on neuroimaging methods and statistical analysis. Any study with a score < 5 was excluded from this review. Additionally, the Grading of Recommendations Assessment, Development, and Evaluation (GRADE) method was used to assess the certainty of the evidence for the defined primary outcomes (Hultcrantz et al. 2017). Two researchers (H.M., B.L.) independently conducted these assessments, and inconsistencies were discussed until a consensus was reached by involving a third reviewer (Y.L.).

### Data analysis

Two researchers (B.L., H.M.) conducted relevant data analyses and collated the results. The qualitative synthesis summarized the changing characteristics of inflammation-related factors and multimodal neuroimaging between the BD and control groups. Specifically, we outlined the values and ranges of altered peripheral inflammation levels; neuroimaging features (e.g., functional connectivity, cortical thickness, volume, surface area, diffusion, and metabolism); and the strength and significance of peripheral inflammation–brain correlations. Moreover, the differences between and limitations of the research characteristics and findings were assessed across multiple clinical dimensions and compared to explore heterogeneity among the study results and its possible causes. A quantitative data synthesis was initially planned but was not conducted due to the high heterogeneity of outcomes between studies and the incomparability of the data collection and analysis methods.

## Results

### Search results and study characteristics

**Fig. 1:**
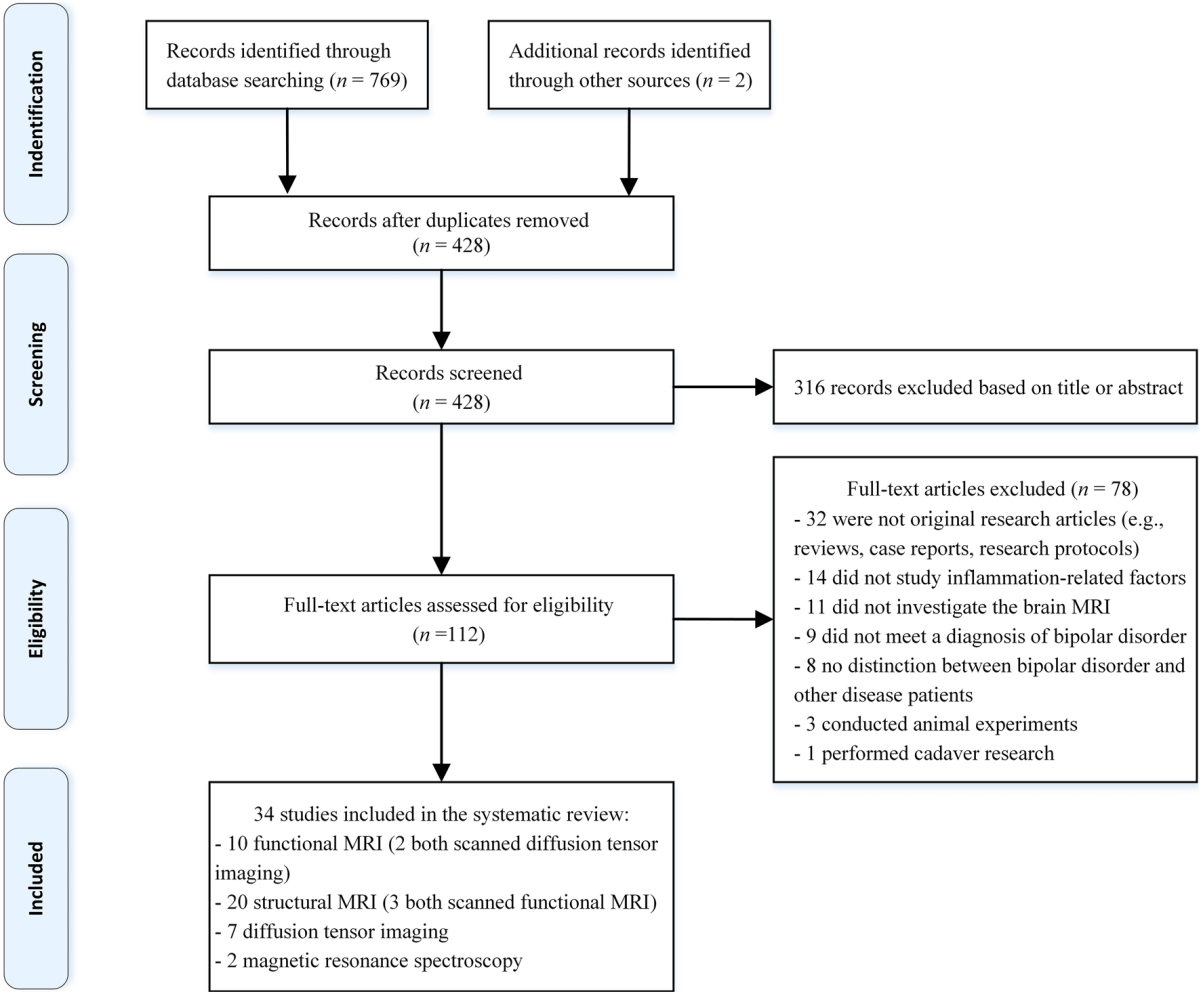
Flowchart of the selection process

A total of 34 studies were included in this review (Chen et al. 2019, 2020b; Tsai et al. 2019; Poletti et al. 2019; Tseng et al. 2021; Mansur et al. 2020; Inal-Emiroglu et al. 2015; Papiol et al. 2008; Chung et al. 2013; Lotrich et al. 2014; Barzman et al. 2014; Savitz et al. 2015; Benedetti et al. 2016a, b; Hoseth et al. 2016; Tu et al. 2017; Besga et al. 2017; Lesh et al. 2018; Furlan et al. 2019; King et al. 2019; Shonibare et al. 2020; Bond et al. 2020; Bai et al. 2020; Quidé et al. 2021; Tang et al. 2021; Mohite et al. 2021; Strenn et al. 2021; Comai et al. 2022; Gong et al. 2022; Jiang et al. 2022) (additional details can be found in Table 1). Twenty-four of the 34 studies enrolled both BD patients and healthy controls (HCs), and ten did not include healthy controls. Six of the 24 studies recruited three groups of subjects: two studies (Chen et al. 2020a; Savitz et al. 2015) included patients with BD, patients with major depressive disorder (MDD), and HCs; one study compared BD patients with suicide attempts, BD patients without suicide attempts, and HCs (Jiang et al. 2022); two studies (Hoseth et al. 2016; Lesh et al. 2018) included BD, schizophrenia, and HC groups; and one study enrolled BD patients, Alzheimer's disease patients, and HCs (Besga et al. 2017). Of the ten studies that did not recruit HCs, seven included only patients with BD (Tsai et al. 2019; Mansur et al. 2020; Chung et al. 2013; Barzman et al. 2014; Benedetti et al. 2016a, b; Tu et al. 2017), and three included BD patients and MDD patients (Chen et al. 2019; Bai et al. 2020; Comai et al. 2022). The literature search and selection process are shown in the flowchart (Fig. 1).

**Table 1:** Summary of characteristics of the included studies

References	BD type	Mood state	Sample size BD/HC	Age (years) BD/HC	Male% BD/HC	Inflammation factors	MRI modality	Outcomes
Benedetti et al. (2016a, b)	I	Depressed	26	47.59	26.9%	SCF	rs-fMRI	Increased stem cell factor levels positively correlated with greater gray matter volume in frontal and parietal cortices and neural responses of ACC and medial PFC
			NA*	NA	NA	BDNF	sMRI	
Benedetti et al. (2016a, b)	I	Depressed	31	48.0	32.0%	TNF-α	DTI	The TNF-α, IFN-γ, IL-8, and IL-10 shared the same associations with lower fractional anisotropy and higher mean diffusivity in the anterior part of brain
			NA	NA	NA	IFN-γ		
Poletti et al. (2017)	I	Depressed	25	47.44	32.0%	Th17 cells	ts-fMRI	The frequency of circulating T regulatory cells correlated to lower neuronal responses in the right dorsolateral PFC and higher radial and mean diffusivity
			21	27.61	34.8%		DTI	
Poletti et al. (2019)	I	Depressed	26	47.96	35.0%	CCL4	rs-fMRI	CCL4 and ICAM1 negatively correlated with cortical thickness in the inferior temporal gyrus; TNF-α positively associated with cortical thickness in the ACC
			23	27.50	34.8%	TNF-α	sMRI	
Chung et al. (2013)	I	Euthymic	17	31.3	41.2%	hs-CRP	sMRI	Elevation of serum hs-CRP levels associated with reduced volume of the orbitofrontal cortex
			NA	NA	NA			
Tsai et al. (2019)	I	Euthymic	32	61.2	37.5%	sIL-2R	sMRI	The bilateral hippocampal volumes were inverse to the sIL-2R level; the total gray matter volume was inverse to sTNF-R1 and IL-1β
			NA	NA	NA	sTNF-R1		
Mohite et al. (2021)	I	Euthymic	21	33.90	33.3%	INF-γ	sMRI	The lower right medial orbitofrontal volume negatively correlated with INF-γ levels; the smaller left posterior cingulate cortex positively correlated with IL-10
			22	33.91	40.9%	IL-10		
Barzman et al. (2014)	I	NA	15	15.0	33.3%	TNF-related gene	ts-fMRI	Expression of TNF-related genes correlated with activation of the amygdala, subgenual ACC, orbitofrontal cortex and thalamus in the affective task
			NA	NA	NA			
Lesh et al. (2018)	I	NA	16	21.4	75.0%	IL-1β, IL-2	sMRI	Whole brain white matter percentage showed an inverse trend with IL-1β
			53	19.5	64.0%			
Chen et al. (2020a)	I	NA	22	28.1	18.2%	TNF-R1	sMRI	The elevated TNF-R1 levels and lower Wisconsin card sorting test performance were positively correlated with smaller gray matter volume in the medial PFC
			22	27.4	18.1%	sIL-6R		
Furlan et al. (2019)	I	Depressed, manic	30	44.23	30.0%	Natural killer	ts-fMRI	The levels of NK cell positively correlated with fractional anisotropy
			36	41.75	33.3%	cells	DTI	
Quidé et al. (2021)	I	Depressed, manic, euthymic, mixed	52	36.43	32.7%	CRP, IL-6	sMRI	Increased systemic inflammation correlated with increased covariation in the striatum and cerebellum
			59	36.27	44.9%			
Chen et al. (2020a)	II	Depressed	41	26.71	43.9%	IL-6	rs-fMRI	The FC between the right posterior insula and left postcentral gyrus was decreased and negatively correlated with the IL-6 level
			68	31.27	48.5%	TNF-α		
Tang et al. (2021)	II	Depressed	42	26.95	45.2%	IL-6	rs-fMRI	The decreased FC of the right postcentral gyrus was inversely correlated with higher IL-8 levels
			69	31.48	47.8%	IL-8		
King et al. (2019)	II	Euthymic	15	22–57	NA	CRP	MRS	There was no significant difference in neuroimaging or inflammatory markers between BD and HC groups
			13	22–57	NA	IL-6		
Savitz et al. (2015)	I, II	Depressed	63	38.8	19.0%	Kynurenic acid	sMRI	The kynurenic acid/3-hydroxy-kynurenine was positively associated with hippocampal and amygdala volume
			48	32.6	40.0%			
Mansur et al. (2020)	I, II	Depressed	55	44.85	20.0%	TNF-α	sMRI	The decreased TNF-α levels were related to increased whole-brain cortical thickness in the infliximab BD group
			NA	NA	NA			
Lotrich et al. (2014)	I, II	Euthymic	21	64.8	38.1%	IL-1RA	DTI	Higher IL-1RA levels inversely correlated with cognitive function even when co-varying for either IL-6 or brain-derived neurotrophic factor
			25	65.5	46.1%	IL-6		
Tu et al. (2017)	I, II	Euthymic	75	42.67	36.0%	sIL-6R	rs-fMRI	Increased sIL-6R correlated with the thinner right middle temporal gyrus cortex
			NA	NA	NA		sMRI	
Emiroglu et al. (2015)	NA	Euthymic	30	16.37	40.0%	NGF	sMRI	The right hippocampal volume negatively correlated with the duration of the disorder and medication, which positively correlated with the nerve growth factor
			23	16.30	52.1%	BDNF		
Tseng et al. (2021)	NA	Euthymic	25	36.48	36.0%	hs-CRP	rs-fMRI	Enhanced FC between the right dorsal caudal putamen and ventrolateral PFC was correlated with higher hs-CRP levels and lower continuous performance tests
			43	31.05	48.8%			
Hoseth et al. (2016)	I, II, NA	NA	117	32.0	39.3%	sTNF-R1	sMRI	Levels of sTNF-R1 were negatively associated with memory learning; hippocampal volume positively correlated with delayed free recall
			236	35.0	44.1%			
Shonibare et al. (2020)	I, II, NA	NA	38	17.5	39.0%	IL-1β genetic variation	sMRI	T-carriers with BD had more significant surface areas in the dorsolateral PFC and lateral occipital cortex
			32	16.4	41.0%			
Strenn et al. (2021)	I, II, NA	NA	188	38.0	39.4%	IL-1β gene	sMRI	The T allele at rs16944 and the C allele at rs1143627 were associated with increased volumes of the left putamen and hippocampus
			54	40.0	40.7%			
Jiang et al. (2022)	I, II, NA	Depressed, manic, euthymic	38	26.46	47.4%	IL-1β	DTI	Fractional anisotropy decreased in the body and genu of the corpus callosum; no significant correlation between inflammation and white matter integrity
			26	28.23	38.5%	TNF-α		
Poletti et al. (2020)	I, II	Depressed, manic, euthymic	63	46.81	27.0%	IL-9	MRS	IL-9 positively predicted glutamate, IL-1β positively predicted Myo-inositol, and CCL5 positively predicted N-acetyl aspartate concentrations
			49	33.70	57.1%	CCL5		
Gong et al. (2022)	I, II	Depressed euthymic	51	27.84	47.1%	TNF-α	rs-fMRI	The higher TNF-α level negatively correlated with decreased FC between the right medial amygdala and bilateral medial frontal cortex
			69	31.48	47.8%	IL-1β		
Chen et al. (2019)	NA	Depressed, manic, euthymic	23	41.96	21.7%	IL-6	sMRI	Proinflammatory cytokines, especially IL-6, were related to the decreased gray matter volume in the frontal, temporal, and cingulate cortex
			NA	NA	NA	TNF-R1		
Bai et al. (2020)	NA	Depressed, manic, euthymic	72	39.5	37.5%	sIL-6R	sMRI	The reduced gray matter volume in 12 brain regions negatively correlated with sIL-6R and sTNF-R1 levels
			NA	NA	NA	sTNF-R1		
Bond et al. (2020)	NA	Depressed, manic, euthymic	51	22.9	47.1%	Epidermal growth factor	sMRI	More mood episodes in the past predicted lower mean log(e)-transformed epidermal growth factor and smaller bilateral temporal lobe volumes
			22	25.0	40.9%			
Bond et al. (2022)	NA	Depressed, manic, euthymic	25	23.0	40.0%	Total inflammation	sMRI	The elevated total inflammation level predicted lower white matter volume in the left frontal lobe and bilateral temporal lobes
			14	24.0	28.6%			
Besga et al. (2017)	NA	NA	24	> 50	NA	IL-1β	DTI	Late-onset BD showed increased IL-1β, IL-6 and fractional anisotropy than Alzheimer’s subjects; IL-6 level positively correlated with white matter integrity
			19	NA	NA	IL-6		
Comai et al. (2022)	NA	NA	66	47.58	33.3%	Kynurenine tryptophan	DTI	Higher kynurenine/tryptophan ratio and IL-1β correlated with lower fractional anisotropy in the inferior fronto-occipital fasciculus
			NA	NA	NA			
Papiol et al. (2008)	NA	NA	20	43.3	50.0%	IL-1β gene (2q13)	sMRI	A polymorphism (rs16944) of the IL-1β gene correlated with whole brain and left dorsolateral PFC gray matter deficits
			43	29.4	55.8%			

BD, bipolar disorder; MRI, magnetic resonance imaging; sMRI, structural MRI; DTI, diffusion tensor imaging; ts-fMRI, task state functional MRI; rs-fMRI, rest state functional MRI; MRS, magnetic resonance spectroscopy; IL, interleukin; sIL-R, soluble IL-receptor; IL-1RA, IL-1 receptor antagonist; hs-CRP, high-sensitivity C-reactive protein; TNF, tumor necrosis factor; TNF-R1, TNF-receptor 1; IFN, interferon; CCL, C–C chemokine ligand; ICAM1, intercellular adhesion molecule; FC, functional connectivity; ACC, anterior cingulate; PFC, prefrontal cortex

*NA: not applicable

### Characteristics of the study populations

Regarding the sex composition of BD patients, all studies except one (Papiol et al. 2008) included more female than male patients. The average age range of BD patients was 16.37 (Inal-Emiroglu et al. 2015)–64.8 (Lotrich et al. 2014) years, with the youngest patient being 12 years old (Chen et al. 2020a) and the oldest patient being 74 years old (Lotrich et al. 2014). Eighteen studies recruited juveniles and adults (Chen et al. 2020a; Tsai et al. 2019; Mansur et al. 2020; Inal-Emiroglu et al. 2015; Chung et al. 2013; Barzman et al. 2014; Tu et al. 2017; Lesh et al. 2018; Poletti et al. 2020; Shonibare et al. 2020; Tang et al. 2021; Mohite et al. 2021; Strenn et al. 2021; Comai et al. 2022; Gong et al. 2022; Jiang et al. 2022; Bond et al. 2022), including three that specifically focused on children and/or adolescent subjects (Inal-Emiroglu et al. 2015; Barzman et al. 2014; Shonibare et al. 2020). Sixteen studies enrolled only adult patients, including two that specifically investigated patients older than 50 years (Lotrich et al. 2014; Besga et al. 2017).

### Clinical characteristics of patients

Excluding three studies (Poletti et al. 2019; Barzman et al. 2014; Besga et al. 2017), 31 described whether patients received therapy before and during the research period. Two of the 31 studies recruited medication-naïve subjects or BD patients who were nonmedicated for at least 6 months (Chen et al. 2020a; Tang et al. 2021); two performed nonpharmacological treatments, one using total sleep deprivation and morning light therapy (Benedetti et al. 2016a, b) and the other using cognitive behavioural therapy (King et al. 2019); the remaining 27 studies used BD-related medications, including lithium, lamotrigine, sodium valproate, antipsychotics, and antidepressants. One study (Mansur et al. 2020) reported that BD patients were taking infliximab (a TNF-α antagonist) to improve depressive symptoms; the remaining studies reported that patients had not taken any immunomodulators or anti-inflammatory drugs in the past 4 weeks.

Twelve studies recruited only BD-I patients: four (Poletti et al. 2017, 2019; Benedetti et al. 2016a, b) focused on patients in the depressed state, three (Tsai et al. 2019; Chung et al. 2013; Mohite et al. 2021) enrolled euthymic subjects, and five (Chen et al. 2020a; Barzman et al. 2014; Lesh et al. 2018; Furlan et al. 2019; Quidé et al. 2021) enrolled patients with unspecified episodes (i.e., different mood states mixed without further classification or no specified description of mood state). Three studies enrolled only patients with BD-II: two (Chen et al. 2020a; Tang et al. 2021) enrolled patients in the depressed state, and one (King et al. 2019) enrolled patients in the euthymic state. The remaining 19 studies involved unspecified BD types (i.e., different types were mixed without further classification or no specified description of subtype): two (Mansur et al. 2020; Savitz et al. 2015) studied patients in the depressed state, four (Tseng et al. 2021; Inal-Emiroglu et al. 2015; Lotrich et al. 2014; Tu et al. 2017) studied patients in the euthymic state, and thirteen (Papiol et al. 2008; Hoseth et al. 2016; Besga et al. 2017; Chen et al. 2019; Poletti et al. 2020; Shonibare et al. 2020; Bond et al. 2020, 2022; Bai et al. 2020; Strenn et al. 2021; Comai et al. 2022; Gong et al. 2022; Jiang et al. 2022) studied patients with unspecified episodes.

### Neuroimaging methods employed in the reviewed studies

Among the 34 studies, 20 utilized structural MRI to evaluate intracranial volume, grey matter volume, white matter volume, cortical thickness, surface area, and hippocampal volume through voxel-based morphometry or surface-based morphometry methods; 7 utilized resting-state fMRI; 3 utilized task-state MRI; 7 utilized DTI to measure the white matter microstructure features of fractional anisotropy, axial diffusivity, radial diffusivity, mean diffusivity, and white matter hyperintensity burden; and 2 utilized magnetic resonance spectroscopy to quantify metabolite concentrations of N-acetyl aspartate (NAA), glutamate (Glu), glutamine (Gln), myoinositol (mI), and creatine (Cr) in the brain. In total, 3 studies collected both resting-state fMRI and structural MRI data; 2 studies collected both DTI and task-state MRI data; and no study used arterial spin labelling or three MRI modalities.

### Inflammation-related factors measured in the reviewed studies

Except for one (Shonibare et al. 2020) study in which saliva samples were collected, the remaining 33 studies assessed inflammation-related factors by collecting peripheral serum or plasma from subjects, and 15 of the 33 studies collected samples between 07:00 a.m. and 1:00 p.m. under fasting conditions. The measured indicators included CRP, hs-CRP, IL, soluble IL-receptor (sIL-R), IL-1β gene polymorphisms, interferon (IFN)-γ, TNF, TNF-receptor 1 (TNF-R1), natural killer (NK) cells, Th17 cells, chemokine C–C motif ligand (CCL), brain-derived trophic factor (BDNF), nerve growth factor (NGF), stem cell factor (SCF), epidermal growth factor (EGF), platelet-derived growth factor (PDGF), kynurenine acid (KynA), tryptophan (Trp), quinolinic acid (QA), and 3-hydroxy-kynurenine (3HK).

### Peripheral inflammation-brain interactions in different clinical dimensions

#### Bipolar I disorder

Depressed BD-I patients. The structural MRI findings were as follows: (1) Benedetti et al. reported that higher stem cell factor levels were related to grey matter volume in the frontal and parietal cortices (Benedetti et al. 2016a, b). (2) Poletti (Poletti et al. 2019) demonstrated that thinner cortical thickness was positively correlated with TNF-α, IL-8, and CCL2 levels and that IL-2R-α levels were inversely linked to cortical thickness in the right parahippocampal gyrus and right inferior temporal gyrus. The DTI findings were as follows: (1) IL-8, IL-10, TNF-α, IFN-γ, and platelet-derived growth factor-BB levels were significantly associated with reduced integrity of white matter microstructure in the cingulum, internal capsule, thalamic radiation, and superior and inferior longitudinal fasciculi (Benedetti et al. 2016a, b). (2) The serum circulating T-cell frequency was positively associated with higher radial diffusivity and mean diffusivity in the right dorsolateral PFC (Poletti et al. 2017). The fMRI findings were as follows: (1) Higher SCF levels covaried with activity in the anterior cingulate cortex and medial PFC (Benedetti et al. 2016a, b). (2) The serum circulating T-cell frequency was positively associated with lower neuronal responses in the right dorsolateral PFC (Poletti et al. 2017). (3) Hypoactivity in the right anterior cingulate cortex was positively correlated with TNF-α, IL-8, and CCL2 levels, while TNF-α and CCL4 levels were negatively associated with the response in the bilateral dorsomedial PFC (Poletti et al. 2019).

Euthymic BD-I patients. The structural MRI findings were as follows: (1) The right hippocampal volume was shown to be adversely related to peripheral sTNF-R1 and soluble IL-2 receptor (sIL-2R) levels, and whole-brain grey matter volume was shown to be negatively correlated with IL-1 and soluble TNF receptor-1 (sTNF-R1) levels (Tsai et al. 2019). (2) Reduced orbitofrontal cortex volume was inversely linked to increased hs-CRP levels, which implicated cognitive impairment (Chung et al. 2013). (3) Reduced medial orbital cortex volume was inversely correlated with IFN-γ levels, while left posterior cingulate cortex volume was positively correlated with IL-10 levels (Mohite et al. 2021).

Patients with unspecified episodes. The structural MRI findings were as follows: (1) Decreased medial PFC volume and executive function were positively associated with increased TNFR1 levels (Chen et al. 2020a). (2) Systemic peripheral inflammation upregulation increased grey matter volume covariance in the striatum and cerebellum (Quidé et al. 2021). (3) 7here was a trend for an inverse relationship between percent whole brain white matter and IL-1β (Lesh et al. 2018). The DTI findings were as follows: (1) NK cell levels positively correlated with fractional anisotropy (Furlan et al. 2019). The fMRI findings were as follows: (1) Patients on long-term lithium therapy had higher NK cell levels and improved cortico-limbic system responses to emotion (Furlan et al. 2019). (2) The expression of 11 TNF-related genes was significantly correlated with activation of the amygdala or the subgenual anterior cingulate cortex in the affective task (Barzman et al. 2014).

#### Bipolar II disorder

Depressed BD-II patients. The fMRI findings were as follows: (1) Increased TNF-α and IL-6 levels were inversely associated with FC between the left postcentral gyrus and the right posterior insula (Chen et al. 2020a). (2) Diminished functional connectivity to the postcentral gyrus was negatively correlated with peripheral inflammation levels (Tang et al. 2021).

Patients in the remission state. The MRS findings were as follows: (1) King (King et al. 2019) et al. analysed the relationships among metabolism in the anterior cingulate cortex, inflammation, and cognitive function, but no significant results were found.

#### Unspecified BD types

Patients in the depressed state. The structural MRI findings were as follows: (1) Lower TNFR1 levels were positively correlated with remission of depressive symptoms and increased cortical thickness of the whole brain after patients received infliximab medication (Mansur et al. 2020). (2) Savitz et al. confirmed that the KynA/QA ratio was significantly lower in BD patients than in healthy controls, and the KynA/3HK ratio was positively correlated with descending hippocampal volume in patients compared with healthy controls (Savitz et al. 2015).

Patients in the remission state. The structural MRI findings were as follows: (1) Inal-Emiroglu revealed that illness duration and treatment with medication were positively associated with NGF and BDNF levels and hippocampal volume (Inal-Emiroglu et al. 2015). (2) Increased sIL-6R levels correlated with a thinner right middle temporal gyrus cortex. The DTI findings were as follows: (1) Elderly BD patients exhibited higher serum IL-1 receptor antagonist (IL-1RA) levels and lower white matter fractional anisotropy values, which were positively correlated with cognitive impairment (Lotrich et al. 2014). The fMRI findings were as follows: (1) Enhanced functional connectivity between the right dorsal caudal putamen and ventrolateral PFC was strongly associated with higher hs-CRP levels (Tseng et al. 2021). (2) Elevated soluble interleukin-6 receptor (sIL-6R) levels were significantly correlated with enhanced functional connectivity between the medial PFC and amygdala and attenuated functional connectivity between the medial PFC and anterior cingulate cortex (Tu et al. 2017).

Patients with unspecified episodes. The structural MRI findings were as follows: (1) Polymorphisms of the IL-1β gene were related to grey matter deficits in the left dorsolateral PFC and the whole brain (Papiol et al. 2008). (2) sTNF-R1 levels had a moderate negative association with performance on memory learning and recall tests, and hippocampal volume had a significant positive correlation with learning list and delayed free recall (Hoseth et al. 2016). (3) Reduced grey matter volume in the orbitofrontal cortex, medial PFC, and inferior frontal cortex were negatively correlated with soluble IL-6R levels (Chen et al. 2019). (4) The dorsolateral PFC and lateral occipital cortex had greater surface areas in T-carriers with BD (Shonibare et al. 2020). (5) More mood episodes in the past predicted lower mean log(e)-transformed epidermal growth factor levels and smaller bilateral temporal lobe volumes (Bond et al. 2020). (6) Smaller volumes of the putamen, hippocampus, postcentral gyrus, and praecuneus negatively correlated with increased IL-6R and sTNF-R1 levels (Bai et al. 2020). (7) The T allele of rs16944 and the C allele of rs1143627 were related to a larger left putamen volume (Strenn et al. 2021). (8) Elevated total peripheral inflammation levels predicted reduced white matter volume in the frontal and temporal lobes in patients with early BD (Bond et al. 2022). The DTI findings were as follows: (1) The inflammatory effects of IL-1β and IL-6 were more pronounced in late-onset BD patients, and IL-6 was positively correlated with white matter integrity (Besga et al. 2017). (2) Increased IL-1β levels and Kyn/Trp ratios were positively associated with decreased FA values in white matter (Comai et al. 2022). (3) BD patients who had attempted suicide had elevated IL-6 levels and significantly lower fractional anisotropy values (Jiang et al. 2022). The fMRI findings were as follows: (1) Elevated TNF-α levels were negatively correlated with decreased FC between the left medial amygdala and left temporal pole (Gong et al. 2022). The MRS findings were as follows: (1) Aberrant concentrations of myoinositol, glutamate, glutathione, and NAA in the anterior cingulate cortex were influenced by IL-1β, IL-9, and CCL5 levels (Poletti et al. 2020).

### Quality assessment

The quality scores for all the studies are shown in Additional file 2: Table S1. Methodological quality assessment according to the STROBE checklist showed that the quality scores of all included studies ranged from 5.0 to 9.0; the overall median and mean scores were 8.0 and 7.93, respectively; and the majority of the included studies received moderate-high scores. These results indicated that there was no serious risk of bias. In addition, the overall certainty of the evidence was rated as "moderate" according to the GRADE assessment criteria (available in Additional file 2: Table S2); “imprecision” was the main reason for the downgrading of the GRADE evaluation. As mentioned above, this systematic review is not appropriate for conducting quantitative analyses. Although further investigations are required to provide high-quality evidence to pinpoint specific inflammation-related factors correlated with BD brain alterations, we identified some interaction patterns in this review and will interpret these qualitative trends below.

## Discussion

**Fig. 2:**
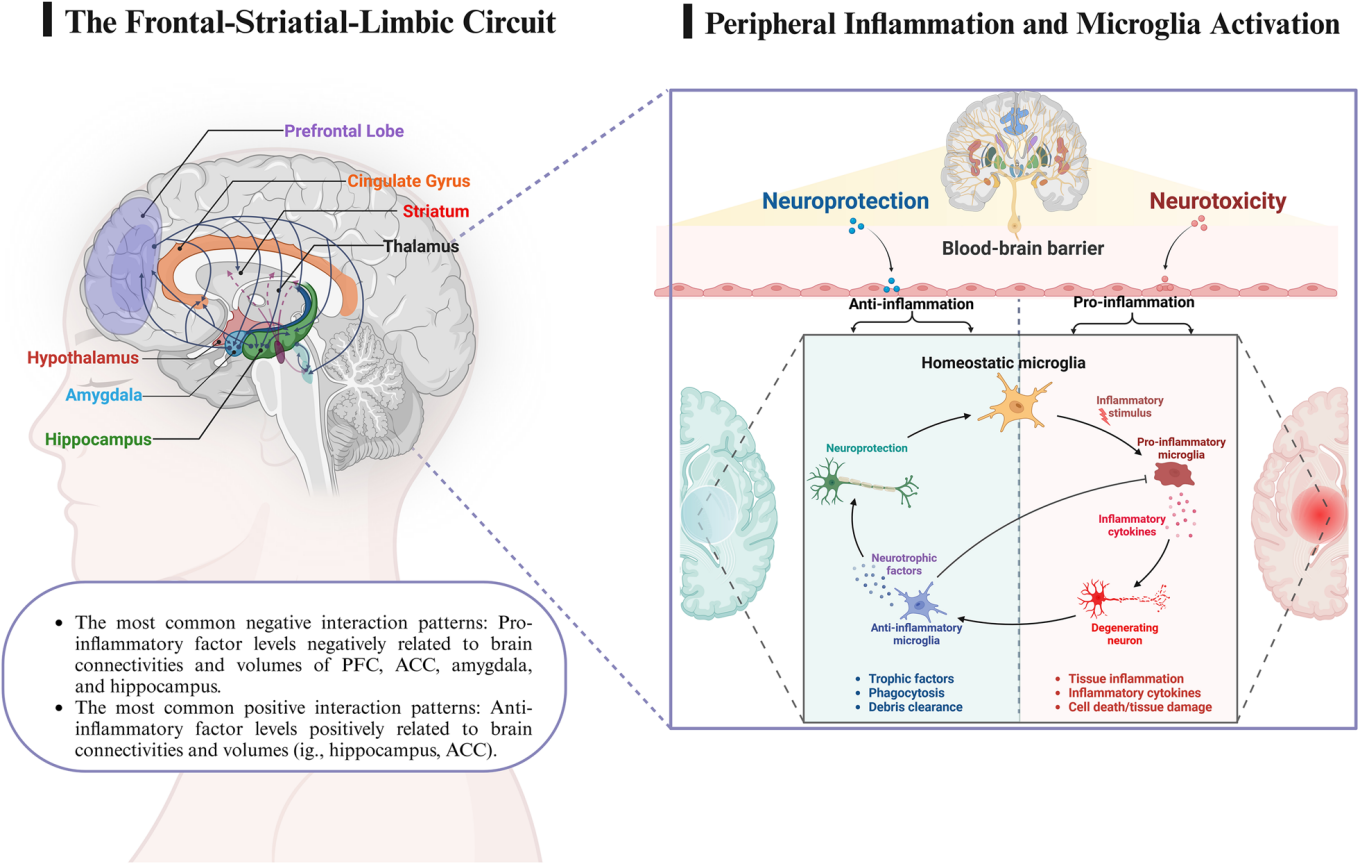
Schematic representation of peripheral inflammation–brain interactions in BD patients. Several inflammatory-related factors can penetrate the blood‒brain barrier and trigger neuroprotective or neurotoxic effects, causing alterations in brain function, the connectome, morphology, microstructure, and metabolism. The PFC, anterior cingulate cortex, amygdala, striatum, and hippocampus are the most vulnerable regions in BD patients, and the neural circuits formed by these areas are essential for emotional processing

The links between peripheral inflammation and neuroimaging findings are intricate and intriguing, and they exhibit a certain degree of heterogeneity and consistency across multiple clinical dimensions (briefly summarized in Fig. 2). To describe these interactions more clearly, we defined them as negative or positive patterns based on covariations between peripheral inflammation and the brain. The following discussion will explain our findings and their implications from broader clinical perspectives.

### Negative and positive peripheral inflammation–brain interaction patterns

#### Negative interaction patterns

The negative correlations between peripheral inflammatory factor levels and the brain changes, which we call negative interaction patterns. Twenty-one studies reported 45 significant negative patterns. Among these, 44 negative patterns consistently indicated that increased peripheral inflammation levels, IL-1β gene polymorphisms (Papiol et al. 2008; Strenn et al. 2021), and TNF gene expression (Barzman et al. 2014) were positively correlated with reduced functional activity, functional connectivity, fractional anisotropy, white matter integrity, white matter volume, grey matter volume, hippocampal volume, and cortical thickness; another negative pattern indicated that declining TNF-α levels were positively correlated with increasing whole-brain cortical thickness (Mansur et al. 2020). Overall, the most common inflammation-related factor changes in these negative patterns were elevated levels of TNF-α, TNF-R1, sTNF-R1, CRP, hs-CRP, IL-1, IL-1β, IL-6, sIL-6R, sIL-2R, IL-8, IL-10, ICAM1, Th17 cells, CCL-4, and CCL-5. The most susceptible brain regions in negative patterns included the dorsolateral PFC, medial PFC, amygdala, anterior cingulate cortex, hippocampus, orbitofrontal cortex, postcentral gyrus, parahippocampal gyrus, superior frontal gyrus, putamen, caudate, fusiform gyrus, and cerebellum, with negative volume changes being the most common. Consistent with our findings, previous laboratory research and clinical observations have confirmed that inflammatory activation is associated with brain disconnection, grey matter loss or atrophy, and disruption of myelin sheath integrity. Generally, this neurological damage stems from the neurotoxic effects of inflammatory activation (Agarwal et al. 2022). Sustained release of proinflammatory factors, cytokines, and chemokines can stimulate neuroglia (especially microglia and astrocytes) (Haarman et al. 2016); conversely, activated glia provoke further inflammatory responses; through increased oxidative stress, apoptotic signalling cascades induce neuronal and glial damage or apoptosis, aberrant synaptic transmission, and impaired myelin production (Kato 2019). In addition, some oversecreted proinflammatory factors can penetrate the blood‒brain barrier, triggering anomalous neuroinflammation procedures, including specific areas and neural circuits related to emotional regulation (Raison et al. 2006), such as the anterior cingulate cortex, PFC, amygdala, and hippocampus.

Our review suggested that the brain regions most susceptible to inflammation associated with negative interaction patterns primarily include the limbic system and PFC. The centrality of the limbic system in emotion processing and regulation is well established, and abnormalities in this system have been broadly reported in previous research on BD (Wang et al. 2016; Long et al. 2022; Lei et al. 2023). Similarly, impairment of the PFC typically leads to abnormal emotion regulation, executive control, and behavioural management (Poletti et al. 2017, 2019; Savitz et al. 2013), and inflammatory activation in the PFC is an important pathophysiological feature of BD (Roman et al. 2021). These findings collectively suggest that inflammatory activation in BD patients can trigger brain alterations and emotion dysregulation. As mentioned above, after receiving anti-inflammatory treatment (the TNF antagonist infliximab), BD patients exhibited reduced peripheral inflammation levels, while whole-brain cortical thickness tended to increase (Mansur et al. 2020). This beneficial negative pattern is consistent with the findings of previous studies, further corroborating that as inflammatory activation decreased, the neurotoxic effects of inflammatory activation on the brain could be partially reversed, and affective symptoms improved (Tseng et al. 2021; Pender 2020). In conjunction with the mild immune activation commonly observed in BD individuals and the amelioration of symptoms after anti-inflammatory therapy, we suggest that monitoring inflammation levels and maintaining immune homeostasis have important clinical implications for diagnosing and treating BD.

#### Positive interaction patterns

In this paper, positive interaction patterns refer to simultaneous increases or decreases in peripheral inflammation and neuroimaging measurements. Fourteen previous studies revealed 20 significant positive patterns, and the majority of these patterns demonstrated concurrent elevation of inflammation-related factors with neuroimaging measurements. The anterior cingulate cortex, hippocampus, amygdala, putamen, medial PFC, pallidum, thalamus, dorsolateral PFC, ventrolateral PFC, and posterior cingulate cortex were the regions with the most significant changes, and altered inflammation-related factors included BDNF, NGF, SCF, EGF, NK cells, IL-1β, IL-6, sIL-6R, IL-9, IL-10, CCL-5, TNF-α, hs-CRP, Kyn and, QA.

The contrasting effects of positive and negative patterns can be interpreted in three ways. (1) Inflammatory mediators possess specific neurosupportive properties (Tseng et al. 2021; Tu et al. 2017; Poletti et al. 2020). For example, IL-1β, TNF-α and CCL5 can stimulate the generation of a reactive astrocyte phenotype, promoting axonal growth, neuronal function enhancement, and microstructural remodelling (Hyvärinen et al. 2019). (2) The compensatory immunomodulatory effects of anti-inflammatory cytokines (Poletti et al. 2017), e.g., IL-10 and NK cells, can compensate for overactivated inflammatory responses and maintain brain homeostasis by protecting neurons and modulating synaptic transmission through multiple pathways (Karthikeyan et al. 2022; Furlan et al. 2019; Maes and Carvalho 2018). (3) The neuroprotective effects of neurotrophic factors, i.e., their participation in neuron–neuron and neuron–glia interactions, facilitate neuronal growth and maintain immune homeostasis (Karthikeyan et al. 2022; Benedetti et al. 2016a, b). In contrast, when epidermal growth factor levels decreased, lower volumes of the brain regions involved in the limbic system were observed in early-stage BD patients. Remarkably, strong evidence from a longitudinal study further indicated an increasing trend in hippocampal volume and neurotrophic factor levels in patients on long-term lithium treatment (Inal-Emiroglu et al. 2015) since lithium can attenuate central nervous system damage and ameliorate affective symptoms through neurotrophic factor-mediated neuroprotection.

In addition, three studies reported positive associations between elevated TNF-α, IL-6, and IL-8 levels and increased mean diffusivity and cortical thickness (Poletti et al. 2019; Benedetti et al. 2016a, b; Besga et al. 2017). These associations may be due to the direct cytotoxic effects of TNF-α on neurons, which induce IL-8 and stimulate expression of cell death mediators, causing intracellular and extracellular oedema and increasing free water molecules, leading to cellular swelling. By continuing to decipher the subtle links among peripheral inflammation, the brain, and behaviour, the neuroprotective effect of peripheral inflammation may be useful for neurological recovery and mood improvement in patients with BD.

### Heterogeneity and consistency of peripheral inflammation–brain interactions

#### Heterogeneity

We found that differences in peripheral inflammation–brain interactions occurred not only in patients with different BD subtypes but also in patients with the same subtypes in different mood states. Compared to changes in peripheral inflammation levels, differences in altered brain areas between subtypes were more pronounced. The hippocampus, parahippocampal gyrus, caudate, medial PFC, anterior cingulate cortex and posterior cingulate cortex were the most common vulnerable regions in BD-I patients, whereas in BD-II patients, alterations in the anterior cingulate cortex, insula, putamen, orbitofrontal cortex, and postcentral gyrus were most common. Similarly, several previous studies have shown specific differences in the abnormal brain regions of patients with different BD clinical subtypes (Liu et al. 2022; Kuang et al. 2021; Ambrosi et al. 2016; Foley et al. 2018). Although the origins of this heterogeneity are still unclear, evidence from integrative genomic analyses indicated distinct aetiological differences between the two subtypes (Huang et al. 2022; Cheng et al. 2021). In other words, several specific neuroimaging characteristics may be potential biomarkers for identifying BD subtypes.

On the other hand, heterogeneity in peripheral inflammation–brain interactions also existed between patients in the euthymic and depressed states. Interestingly, positive interaction patterns were found in a greater proportion of patients in remission than those with depression, i.e., this mood state showed more positive changes. Coincidentally, depressed BD patients demonstrated more severe white matter injuries than euthymic BD patients in an earlier study (Yang et al. 2020). Additionally, FCs between several core areas (e.g., the subgenual anterior cingulate cortex and ventrolateral PFC) were significantly greater in patients in remission than in those in the depressed state (Rey et al. 2016), which is consistent with our findings. We speculate that these positive trends in neuroimaging findings in euthymic BD patients may be caused by several neurorestorative and compensatory mechanisms. Specifically, with the gradual stabilization of the disease (into remission), the function of core areas related to emotional processing is gradually restored. Subsequently, through compensatory self-regulation, the information flow between regions (e.g., the PFC and anterior cingulate cortex) is gradually enhanced, mitigating the adverse effects of the widespread reduction in whole-brain functional connectivity; in parallel, accompanied by the ongoing repair of microstructures, the brain morphology gradually normalizes.

Moreover, differences in the brain regions susceptible to inflammation-related factors were found between patients in the depression state and those in the remission state. Notably, the functional connectivity involving the striatum increased during remission but rarely occurred during depression. As a core node in the fronto-limbic-striatal system, the striatum plays a vital role in cognitive integration and emotional processes; thus, the striatum is crucial in the neurobiology of affective disorders. According to the positive alterations in the striatum, especially the heightened functional connectivity between other emotion-processing regions mostly appearing in remission, and its involvement in several neural circuits, we hypothesize that this phenomenon may represent a gradual recovery of the patient's emotional processing capacity as the condition improves (being in remission). Whether such positive changes in the striatum can be considered a potential neuroimaging phenotype to distinguish the euthymic phase is unclear, and additional studies with larger sample sizes and more comprehensive explorations are needed.

#### Consistency

In addition to heterogeneity, there was some consistency in peripheral inflammation–brain interaction patterns across different clinical dimensions. The most commonly altered inflammation-related markers were TNF-α, TNF-R1, sTNF-R1, CRP, hs-CRP, IL-1β, sIL-2R, IL-6, sIL-6R, IL-8, IL-9, IL-10, IFN-γ, BDNF, and NGF. In addition, the most vulnerable brain regions included the anterior cingulate cortex, amygdala, PFC, orbitofrontal cortex, hippocampus, parahippocampal gyrus, putamen, caudate, pallidum, postcentral gyrus, and posterior cingulate cortex. These findings are highly consistent with the evidence from numerous previous immunological or imaging studies (Chen et al. 2014, 2020b; Karthikeyan et al. 2022; Tsai et al. 2019; Poletti et al. 2019; Savitz et al. 2013; Tseng et al. 2021), providing further evidence to establish potential biomarkers of BD. Remarkably, negative peripheral inflammation–brain interaction patterns were most prevalent across all BD types and mood states; among these patterns, increased inflammation-related factor levels coupled with decreased brain structure, functional connectivity, white matter integrity, and metabolism were the most common. In other words, this form of covariation is the most typical peripheral inflammation–brain interaction pattern in BD, occurring across age brackets and clinical dimensions. Based on typical patterns, developing reliable peripheral inflammation–neuroimaging integrative targets is anticipated to provide a novel pathway to transcend traditional diagnostic paradigms.

### Fronto-limbic-striatal system

Overall, the PFC, anterior cingulate cortex, amygdala, hippocampus, and striatum were the most susceptible regions across multiple neuroimaging modalities and clinical dimensions, highlighting the critical role of the fronto-limbic-striatal system in the neuropathological mechanisms of BD. In many previous studies, brain abnormalities in this system were prevalent and diverse, showing remarkable consistency with our findings (Vulser et al. 2015; Minuzzi et al. 2018; Leibenluft et al. 2003; Lu et al. 2021). This neural system is well known to be critical for inducing emotional dysregulation and cognitive deficits (Leibenluft et al. 2003; Brotman et al. 2014) and is even the principal neural basis for more severe psychotic symptoms in paediatric BD patients (Gao et al. 2021). Coincidentally, administering vagus nerve stimulation to this system can effectively alleviate emotional symptoms (Wang et al. 2021). This result may be caused by the downregulation of neuroinflammatory activation states by physical stimulation, which modulates brain regions involved in autonomic responses (Wei et al. 2022; Gottesman and Gould 2003). Our study further confirmed that this system may be a shared neurobiological substrate for different BD types and mood states.

## Conclusions and limitations

To our knowledge, this is the first systematic review of interactions between peripheral inflammation-related factors and multimodal neuroimaging in different clinical dimensions of BD. The majority of the studies included in this review were of moderate-high quality and investigated multiple BD subtypes and mood states, suggesting that the outcomes are representative of peripheral inflammation–brain relationships. However, there are several limitations. First, although most of the included studies received moderate-high quality assessment scores, a few were of low quality, with a potential risk of bias in several domains. For example, six studies did not recruit healthy controls for comparison, and two did not adequately evaluate and record demographic or clinical information. Although we tried to minimize publication bias by searching for unpublished studies in databases, it is still difficult to eliminate because scholars usually only demonstrate statistically significant results. Second, we included some studies that did not explicitly describe the subtypes or episodes of BD. For example, eight studies did not specify patient subtypes, and eight did not report the current mood states of the patients. Third, the included studies mainly measured brain–peripheral inflammation correlations, which cannot indicate causality. Fourth, because most of the studies were cross-sectional, determining the dynamics of peripheral inflammation-brain interactions was impossible. Finally, the high methodological and clinical heterogeneity between studies prevented quantitative data synthesis or subgroup analysis and hindered the comparison of results. Therefore, larger sample sizes, multicenter studies, and standardized study designs are needed for further investigations to overcome the low statistical power of the existing research.

Despite the above limitations, we identified extensive and complex associations between peripheral inflammation-related factors and multimodal neuroimaging alterations from a clinical perspective and the heterogeneity and consistency of peripheral inflammation–brain interactions across different clinical dimensions. In summary, our proposed novel interaction patterns contribute to a better understanding of the neuropathological mechanisms of BD, and the shared and specific neuroimaging substrates we identified may be potential biomarkers for precision medicine.

### Supplementary Information

**Additional file 1**. Search strategy using keywords according to the databases.

**Additional file 2. Table S1**: Checklist of quality assessment for the included studies in this review. **Table S2**: Certainty of the evidence for the main outcomes.

## Data Availability

The datasets used and/or analysed during the present study are available from the corresponding author on reasonable request.
