# Supplementary material for: Correlations between multimodal neuroimaging and peripheral inflammation in different subtypes and mood states of bipolar disorder: a systematic review
Source: Int J Bipolar Disord. 2024 Feb 22;12:5. doi: 10.1186/s40345-024-00327-w (PMC10884387; doi:10.1186/s40345-024-00327-w)
Supplement: Supplementary file 1 — Additional file 1. Search strategy using keywords according to the databases. [file 40345_2024_327_MOESM1_ESM.pdf]

Material Additional file 1

Search Strategy using keywords as per databases

1. PubMed (Search Hits-89) Filters Applied: Journal Articles, Publication date from the inception of database up to 2023/07/01

(("Bipolar Disorder"[Mesh] OR "Bipolar Disorders" [All Fields] OR "Cyclothymic disorder" [All Fields] OR "Cyclothymia" [All Fields] OR "Disorder, Bipolar" [All Fields] OR "Psychosis, Manic-Depressive" [All Fields] OR "Psychosis, Manic Depressive" [All Fields] OR "Psychoses, Manic-Depressive" [All Fields] OR "Psychoses, Manic Depressive" [All Fields] OR "Manic-Depressive Psychosis" [All Fields] OR "Manic Depressive Psychosis" [All Fields] OR "Bipolar Mood Disorder" [All Fields] OR "Bipolar Mood Disorders" [All Fields] OR "Disorder, Bipolar Mood" [All Fields] OR "Mood Disorder, Bipolar" [All Fields] OR "Affective Psychosis, Bipolar" [All Fields] OR "Bipolar Affective Psychosis" [All Fields] OR "Psychoses, Bipolar Affective" [All Fields] OR "Psychosis, Bipolar Affective" [All Fields] OR "Bipolar Disorder Type 1" [All Fields] OR "Type 1 Bipolar Disorder" [All Fields] OR "Manic Depression" [All Fields] OR "Depression, Manic" [All Fields] OR "Depressions, Manic" [All Fields] OR "Depression, Bipolar" [All Fields] OR "Bipolar Depression" [All Fields] OR "Bipolar Disorder Type 2" [All Fields] OR "Type 2 Bipolar Disorder" [All Fields] OR "Manic Disorder" [All Fields] OR "Disorder, Manic" [All Fields] OR "Manic Disorders" [All Fields]) AND ("Cytokines"[Mesh] OR "Cytokine" [All Fields] OR "Macrophage Inflammatory Proteins" [All Fields] OR "Growth Differentiation Factor" [All Fields] OR "Hematopoietic Cell Growth Factors" [All Fields] OR "Hematopoietic Cell Growth Factors" [All Fields] OR "Colony-Stimulating Factors" [All Fields] OR "Stem Cell Factor" [All Fields] OR "Hepatocyte Growth Factor" [All Fields] OR "Interferons" [All Fields] OR "Interleukins" [All Fields] "OR Interleukin" [All Fields] OR "Leukemia Inhibitory Factor" [All Fields] OR "Lymphokines" [All Fields] OR "Leukocyte Migration-Inhibitory Factors" [All Fields] OR "Leukocyte Migration-Inhibitory Factors" [All Fields] OR "Macrophage-Activating Factors" [All Fields] OR "Macrophage Migration-Inhibitory Factors" [All Fields] OR "Macrophage Migration-Inhibitory Factors" [All Fields] OR "Macrophage Migration-Inhibitory Factors" [All Fields] OR "Monokines" [All Fields] OR "Oncostatin M" [All Fields] OR "Thymic Stromal

Lymphopoietin" [All Fields] OR "Transforming Growth Factor beta" [All Fields] OR "Tumor Necrosis Factor" [All Fields] OR "Immune" [All Fields] OR "Immunity" [All Fields] OR "Neuro-immunomodulation" [All Fields] OR "Neuro immunomodulation" [All Fields] OR "Neuroimmune Processes" [All Fields] OR "Neuro-immune Communication" [All Fields] OR "Communication, Neuro-immune" [All Fields] OR "Neuro immune Communication" [All Fields] OR "Neuro-immune Communications" [All Fields] OR "Neuroimmune Communication" [All Fields] OR "Communication, Neuroimmune" [All Fields] OR "Neuroimmune Communications" [All Fields] OR "Neuroimmune Interactions" [All Fields] OR "Interaction, Neuroimmune" [All Fields] OR "Neuroimmune Interaction" [All Fields] OR "Neuroimmune Process" [All Fields] OR "Process, Neuroimmune" [All Fields] OR "Vagal Anti-inflammatory Pathway" [All Fields] OR "Anti-inflammatory Pathway, Vagal" [All Fields] OR "Anti inflammatory Pathway" [All Fields] OR "Immune Interactions" [All Fields] OR "Neuro immune Axis" [All Fields] OR "Neuroimmune Axis" [All Fields] OR "Cholinergic Anti-inflammatory Pathway" [All Fields] OR "Anti-inflammatory Pathway, Cholinergic" [All Fields] OR "Cholinergic Anti inflammatory Pathway" [All Fields] OR "Cholinergic Anti-inflammatory Pathways" [All Fields])) AND ("Magnetic Resonance Imaging"[Mesh] OR "Magnetic Resonance" [All Fields] OR "NMR Imaging" [All Fields] OR "Imaging, NMR" [All Fields] OR Tomography, NMR" [All Fields] OR "Tomography, MR" [All Fields] OR "MR Tomography" [All Fields] OR "NMR Tomography" [All Fields] OR "Steady-State Free Precession MRI" [All Fields] OR "Steady State Free Precession MRI" [All Fields] OR "Zeugmatography" [All Fields] OR "Imaging, Chemical Shift" [All Fields] OR "Chemical Shift Imagings" [All Fields] OR "Imagings, Chemical Shift" [All Fields] OR "Shift Imaging, Chemical" OR [All Fields] "Shift Imagings, Chemical" [All Fields] OR "Chemical Shift Imaging" [All Fields] OR "Magnetic Resonance Image" [All Fields] OR "Image, Magnetic Resonance" [All Fields] OR "Magnetic Resonance Images" [All Fields] OR "Resonance Image, Magnetic" [All Fields] OR "Magnetization Transfer Contrast Imaging" [All Fields] OR "MRI Scans" [All Fields] OR "MRI Scan" [All Fields] OR "Scan, MRI" [All Fields] OR "Scans, MRI" [All Fields] OR "Tomography, Proton Spin" [All Fields] OR "Proton Spin Tomography" [All Fields] OR "fMRI" [All Fields] OR "MRI, Functional" [All Fields] OR "Functional MRI" [All Fields] OR "Functional MRIs" [All Fields] OR "MRIs, Functional" [All Fields] OR

"Functional Magnetic Resonance Imaging" [All Fields] OR "Magnetic Resonance Imaging, Functional" [All Fields] OR "Spin Echo Imaging" [All Fields] OR "Echo Imaging, Spin" [All Fields] OR "Echo Imagings, Spin" [All Fields] OR "Imaging, Spin Echo" [All Fields] OR "Imagings, Spin Echo" [All Fields] OR "Spin Echo Imagings" [All Fields] OR "Diffusion Tensor" [All Fields] OR "Diffusion Tensor Magnetic Resonance Imaging" [All Fields] OR "Diffusion Tensor MRI" [All Fields] OR "Diffusion Tensor MRIs" [All Fields] OR "MRI, Diffusion Tensor" [All Fields] OR "DTI MRI" [All Fields] OR "Diffusion Tractography" [All Fields] OR "Tractography, Diffusion" [All Fields] OR "Structural Resonance Spectrum" [All Fields] OR "sMRI" [All Fields] OR "Magnetic Resonance Spectrum" [All Fields] OR "MRS" [All Fields] OR "Arterial spin labeling" [All Fields] OR "ASL" [All Fields])

2. Web of Science Core Collection (Search Hits-404) Publication date from the inception of database up to 2023/07/01

TOPIC: (bipolar disorder) OR TOPIC: (bipolar depression) OR TOPIC: (manic depressive) OR TOPIC: (cyclothymic disorder) OR TOPIC: (cyclothymia) AND TOPIC: (inflammation) OR TOPIC: (inflammatory) OR TOPIC: (immune) OR TOPIC: (immunity) OR TOPIC: (cytokines) OR TOPIC: (chemokines) AND TOPIC: (magnetic resonance imaging) OR TOPIC: (functional magnetic resonance imaging) OR TOPIC: (structural magnetic resonance imaging) OR TOPIC: (diffusion tensor imaging) OR TOPIC: (magnetic resonance spectroscopy) OR TOPIC: (arterial spin labeling)

3. Scopus (Search Hits-109) Publication date from the inception of database up to 2023/07/01

TOPIC: (bipolar disorder) OR TOPIC: (bipolar depression) OR TOPIC: (manic depressive) OR TOPIC: (cyclothymic disorder) OR TOPIC: (cyclothymia) AND TOPIC: (inflammation) OR TOPIC: (inflammatory) OR TOPIC: (immune) OR TOPIC: (immunity) OR TOPIC: (cytokines) OR TOPIC: (chemokines) AND TOPIC: (magnetic resonance imaging) OR TOPIC: (functional magnetic resonance imaging) OR TOPIC: (structural magnetic resonance imaging) OR TOPIC: (diffusion tensor imaging) OR TOPIC: (magnetic resonance spectroscopy) OR TOPIC: (arterial spin labeling)

4. EMBASE (Search Hits-102) Publication date from the inception of database up to 2023/07/01TOPIC: (bipolar disorder) OR TOPIC: (bipolar depression) OR TOPIC: (manic depressive) OR TOPIC: (cyclothymic disorder) OR TOPIC: (cyclothymia) AND TOPIC: (inflammation) OR TOPIC: (inflammatory) OR TOPIC: (immune) OR TOPIC: (immunity) OR TOPIC: (cytokines) OR TOPIC: (chemokines) AND TOPIC: (magnetic resonance imaging) OR TOPIC: (functional magnetic resonance imaging) OR TOPIC: (structural magnetic resonance imaging) OR TOPIC: (diffusion tensor imaging) OR TOPIC: (magnetic resonance spectroscopy) OR TOPIC: (arterial spin labeling)

5. MEDLINE (Search Hits-26) Publication date from the inception of database up to 2023/07/01 TOPIC: (bipolar disorder) OR TOPIC: (bipolar depression) OR TOPIC: (manic depressive) OR TOPIC: (cyclothymic disorder) OR TOPIC: (cyclothymia) AND TOPIC: (inflammation) OR TOPIC: (inflammatory) OR TOPIC: (immune) OR TOPIC: (immunity) OR TOPIC: (cytokines) OR TOPIC: (chemokines) AND TOPIC: (magnetic resonance imaging) OR TOPIC: (functional magnetic resonance imaging) OR TOPIC: (structural magnetic resonance imaging) OR TOPIC: (diffusion tensor imaging) OR TOPIC: (magnetic resonance spectroscopy) OR TOPIC: (arterial spin labeling)

6. PsycInFO (Search Hits-31) Publication date from the inception of database up to 2023/07/01

TOPIC: (bipolar disorder) OR TOPIC: (bipolar depression) OR TOPIC: (manic depressive) OR TOPIC: (cyclothymic disorder) OR TOPIC: (cyclothymia) AND TOPIC: (inflammation) OR TOPIC: (inflammatory) OR TOPIC: (immune) OR TOPIC: (immunity) OR TOPIC: (cytokines) OR TOPIC: (chemokines) AND TOPIC: (magnetic resonance imaging) OR TOPIC: (functional magnetic resonance imaging) OR TOPIC: (structural magnetic resonance imaging) OR TOPIC: (diffusion tensor imaging) OR TOPIC: (magnetic resonance spectroscopy) OR TOPIC: (arterial spin labeling)

7. Cochrane Library (Search Hits-8) Publication date from the inception of database up to

2023/07/01

TOPIC: (bipolar disorder) OR TOPIC: (bipolar depression) OR TOPIC: (manic depressive) OR TOPIC: (cyclothymic disorder) OR TOPIC: (cyclothymia) AND TOPIC: (inflammation) OR TOPIC: (inflammatory) OR TOPIC: (immune) OR TOPIC: (immunity) OR TOPIC: (cytokines) OR TOPIC: (chemokines) AND TOPIC: (magnetic resonance imaging) OR TOPIC: (functional magnetic resonance imaging) OR TOPIC: (structural magnetic resonance imaging) OR TOPIC: (diffusion tensor imaging) OR TOPIC: (magnetic resonance spectroscopy) OR TOPIC: (arterial spin labeling)
