# Supplementary material for: Correlations between multimodal neuroimaging and peripheral inflammation in different subtypes and mood states of bipolar disorder: a systematic review
Source: Int J Bipolar Disord. 2024 Feb 22;12:5. doi: 10.1186/s40345-024-00327-w (PMC10884387; doi:10.1186/s40345-024-00327-w)
Supplement: Supplementary file 2 — Additional file 2. Table S1: Checklist of quality assessment for the included studies in this review. Table S2: Certainty of the evidence for the main outcomes. [file 40345_2024_327_MOESM2_ESM.docx]

| **Table S2** The checklist of quality assessment for included studies in this review. | | | | | | | | | | |
| --- | --- | --- | --- | --- | --- | --- | --- | --- | --- | --- |
| **Study** | **1** | **2** | **3** | **4** | **5** | **6** | **7** | **8** | **9** | **Total** |
| Papiol et al., 2008^(1)^ | 0 | 0 | 1 | 1 | 1 | 1 | 1 | 1 | 1 | 7.0 |
| Chung et al., 2013^(2)^ | 1 | 1 | 1 | 1 | 1 | 1 | 1 | 1 | 1 | 9.0 |
| Lotrich et al., 2014^(3)^ | 0.5 | 1 | 1 | 1 | 1 | 1 | 1 | 1 | 1 | 8.5 |
| Barzman et al., 2014^(4)^ | 1 | 0 | 1 | 0 | 0 | 0 | 1 | 1 | 1 | 5.0 |
| Savitz et al., 2015^(5)^ | 0.5 | 0.5 | 1 | 1 | 1 | 1 | 1 | 1 | 1 | 8.0 |
| Emiroglu et al., 2015^(6)^ | 0 | 1 | 1 | 1 | 1 | 1 | 1 | 1 | 1 | 8.0 |
| Benedetti et al., 2016^(7)^ | 1 | 1 | 1 | 1 | 1 | 1 | 1 | 1 | 1 | 9.0 |
| Benedetti et al., 2016^(8)^ | 1 | 1 | 1 | 1 | 1 | 1 | 1 | 1 | 1 | 9.0 |
| Hoseth et al., 2016^(9)^ | 1 | 0 | 1 | 1 | 1 | 1 | 1 | 1 | 1 | 8.0 |
| Poletti et al., 2017^(10)^ | 1 | 1 | 1 | 1 | 1 | 1 | 1 | 1 | 1 | 9.0 |
| Tu et al., 2017^(11)^ | 0.5 | 1 | 1 | 1 | 1 | 1 | 1 | 1 | 1 | 8.5 |
| Besga et al., 2017^(12)^ | 0 | 0 | 0 | 1 | 1 | 0 | 1 | 1 | 1 | 5.0 |
| Lesh et al., 2018^(13)^ | 0.5 | 0 | 1 | 1 | 1 | 1 | 1 | 1 | 1 | 7.5 |
| Poletti et al., 2019^(14)^ | 1 | 1 | 1 | 1 | 0 | 0 | 1 | 1 | 1 | 7.0 |
| Tsai et al., 2019^(15)^ | 1 | 1 | 1 | 1 | 1 | 1 | 1 | 1 | 1 | 9.0 |
| Furlan et al., 2019^(16)^ | 1 | 1 | 1 | 1 | 1 | 1 | 1 | 1 | 1 | 9.0 |
| King et al., 2019^(17)^ | 1 | 1 | 1 | 0 | 0 | 1 | 1 | 1 | 1 | 7.0 |
| Chen et al., 2019^(18)^ | 0 | 0.5 | 1 | 1 | 1 | 1 | 1 | 1 | 1 | 7.5 |
| Chen et al., 2020^(19)^ | 1 | 1 | 1 | 1 | 1 | 1 | 1 | 1 | 1 | 9.0 |
| Mansur et al., 2020^(20)^ | 0.5 | 1 | 1 | 1 | 1 | 1 | 1 | 1 | 1 | 8.5 |
| Poletti et al., 2020^(21)^ | 0.5 | 0.5 | 1 | 1 | 1 | 1 | 1 | 1 | 1 | 8.0 |
| Shonibare et al., 2020^(22)^ | 0.5 | 0 | 1 | 1 | 1 | 1 | 1 | 1 | 1 | 7.5 |
| Bond et al., 2020^(23)^ | 0 | 0.5 | 1 | 1 | 1 | 1 | 1 | 1 | 1 | 7.5 |
| Bai et al., 2020^(24)^ | 0 | 0.5 | 1 | 1 | 1 | 1 | 1 | 1 | 1 | 7.5 |
| Chen et al., 2020^(25)^ | 1 | 0 | 1 | 1 | 1 | 1 | 1 | 1 | 1 | 8.0 |
| Quidé et al., 2021^(26)^ | 1 | 0.5 | 1 | 1 | 1 | 1 | 1 | 1 | 1 | 8.5 |
| Tang et al., 2021^(27)^ | 1 | 1 | 1 | 1 | 1 | 1 | 1 | 1 | 1 | 9.0 |
| Mohite et al., 2021^(28)^ | 1 | 1 | 1 | 1 | 1 | 1 | 1 | 1 | 1 | 9.0 |
| Strenn et al., 2021^(29)^ | 0.5 | 0 | 1 | 1 | 1 | 1 | 1 | 1 | 1 | 7.5 |
| Tseng et al., 2021^(30)^ | 0 | 1 | 1 | 1 | 1 | 1 | 1 | 1 | 1 | 8.0 |
| Comai et al., 2022^(31)^ | 0 | 0 | 1 | 1 | 1 | 1 | 1 | 1 | 1 | 7.0 |
| Gong et al., 2022^(32)^ | 0.5 | 0.5 | 1 | 1 | 1 | 1 | 1 | 1 | 1 | 8.0 |
| Jiang et al., 2022^(33)^ | 0.5 | 0.5 | 1 | 1 | 1 | 1 | 1 | 1 | 1 | 8.0 |
| Bond et al., 2022^(34)^ | 0 | 0.5 | 1 | 1 | 1 | 1 | 1 | 1 | 1 | 7.5 |

Additional file 2: Table S2

**Items of quality assessment**：

1. Were the descriptions of the patient's type of BD clear and adequate? 1= Yes, 0.5= multiple types exist but did not study in subgroups, 0= No or too less information;
2. Were the descriptions of the patient's mood state clear and adequate? 1= Yes, 0.5= multiple states exist but did not study in subgroups, 0= no or too less information, 0= No or too less information;
3. Were the descriptions of the control group clear and adequate? 1= Yes, 0= No or too less information;
4. Were the patient group comparable to the control group in terms of age and gender? 1= Yes, 0= No;
5. Whether all patients were free of comorbidity? (Assessing the presence of other psychiatric disorders, infections or immunological system diseases) 1= Yes, 0= No or too less information;
6. Whether the medication history has been reported? 1= without medication for BD or any medications known to affect the immune system, 0.5= reported or discontinued before MRI scanning, 0= Not reported;
7. Whether the descriptions of the imaging technique were clear so that it could be reproduced? 1= Yes, 0= No;
8. Whether statistical results were corrected for multiple comparison? 1= corrected, 0= uncorrected or not applicable;
9. Whether the conclusions were consistent with the results obtained, and the limitations were discussed? 1= Yes, 0= No.

**Risk of bias assessment of included studies:** Methodological quality assessment through the Strengthening Checklist for Reporting of Observational Studies in Epidemiology (STROBE) showed that the quality scores of all included studies ranged from 5.0-9.0; the overall median and mean scores were 8.0 and 7.93, respectively; and most of the included studies received moderate-high scores. Therefore, no serious risk of bias was identified.

Additional file 2: Table S3

| **Table S3** **Certainty of the evidence of** **main outcomes** | | | | | | |
| --- | --- | --- | --- | --- | --- | --- |
| **Number of participants**  **(Number of Studies)** | **Certainty of the evidence (GRADE)** | | | | | |
|  | **Risk of bias** | **Inconsistency** | **Indirectness** | **Imprecision** | **Publication bias** | **Overall quality** |
| 2,993  (n=34) | Not serious ^a^ | Not serious ^b^ | Not serious ^c^ | Serious ^d^ | Not serious ^e^ | Moderate  ⊕⊕⊕ |

GRADE: Grading of Recommendations Assessment, Development and Evaluation; NA: not applicable.

1. The overall STROBE rating; overall median and mean scores were 8.0 and 7.93, respectively.
2. Inconsistency was judged by assessing the consistency of changing trends in inflammation and neuroimaging and differences in inflammation-neuroimaging correlations across studies (because statistical measures of heterogeneity were not available). As we did not find significant differing results between the outcomes of the included studies, we considered the risk of inconsistency to be "not serious".
3. Approximately 60% of the studies had adequate sample frames to analyze the relevant indicators.
4. Imprecision was judged by evaluating the range of fluctuating inflammation levels, the precise location (coordinate) and specific value of altered brain regions, and the strength and significance of inflammation and neuroimaging correlations. It was downgraded due to approximately 1/3 of the outcomes reported by the included studies were not sufficiently precise and detailed.
5. Because only 2 studies selectively demonstrated a trend towards a particular topic or a particular outcome, the rank of the publication bias domain was considered "not serious".

**GRADE for quality of evidence**: Thirty-four studies included primary outcomes involving fluctuating inflammation-related markers levels, altered multimodal neuroimaging characteristics, and correlations of inflammation and brain in patients with bipolar disorder. The imprecision was the main reason for the downgrading of the GRADE evaluation in this systematic review.

References

1. Papiol S, Molina V, Desco M, Rosa A, Reig S, Sanz J, et al. Gray matter deficits in bipolar disorder are associated with genetic variability at interleukin-1 beta gene (2q13). Genes, brain, and behavior. 2008;7(7):796-801.

2. Chung KH, Huang SH, Wu JY, Chen PH, Hsu JL, Tsai SY. The link between high-sensitivity C-reactive protein and orbitofrontal cortex in euthymic bipolar disorder. Neuropsychobiology. 2013;68(3):168-73.

3. Lotrich FE, Butters MA, Aizenstein H, Marron MM, Reynolds CF, 3rd, Gildengers AG. The relationship between interleukin-1 receptor antagonist and cognitive function in older adults with bipolar disorder. International journal of geriatric psychiatry. 2014;29(6):635-44.

4. Barzman D, Eliassen J, Mcnamara R, Abonia P, Mossman D, Durling M, et al. Correlations of inflammatory gene pathways, corticolimbic functional activities, and aggression in pediatric bipolar disorder: A preliminary study. Psychiatry research. 2014;224(2):107-11.

5. Savitz J, Dantzer R, Wurfel BE, Victor TA, Ford BN, Bodurka J, et al. Neuroprotective kynurenine metabolite indices are abnormally reduced and positively associated with hippocampal and amygdalar volume in bipolar disorder. Psychoneuroendocrinology. 2015;52:200-11.

6. Inal-Emiroglu FN, Resmi H, Karabay N, Guleryuz H, Baykara B, Cevher N, et al. Decreased right hippocampal volumes and neuroprogression markers in adolescents with bipolar disorder. Neuropsychobiology. 2015;71(3):140-8.

7. Benedetti F, Poletti S, Hoogenboezem TA, Locatelli C, Ambrée O, de Wit H, et al. Stem Cell Factor (SCF) is a putative biomarker of antidepressant response. Journal of neuroimmune pharmacology : the official journal of the Society on NeuroImmune Pharmacology. 2016;11(2):248-58.

8. Benedetti F, Poletti S, Hoogenboezem TA, Mazza E, Ambrée O, de Wit H, et al. Inflammatory cytokines influence measures of white matter integrity in Bipolar Disorder. Journal of affective disorders. 2016;202:1-9.

9. Hoseth EZ, Westlye LT, Hope S, Dieset I, Aukrust P, Melle I, et al. Association between cytokine levels, verbal memory and hippocampus volume in psychotic disorders and healthy controls. Acta psychiatrica Scandinavica. 2016;133(1):53-62.

10. Poletti S, de Wit H, Mazza E, Wijkhuijs AJM, Locatelli C, Aggio V, et al. Th17 cells correlate positively to the structural and functional integrity of the brain in bipolar depression and healthy controls. Brain, behavior, and immunity. 2017;61:317-25.

11. Tu PC, Li CT, Lin WC, Chen MH, Su TP, Bai YM. Structural and functional correlates of serum soluble IL-6 receptor level in patients with bipolar disorder. Journal of affective disorders. 2017;219:172-7.

12. Besga A, Chyzhyk D, Gonzalez-Ortega I, Echeveste J, Graña-Lecuona M, Graña M, et al. White Matter Tract Integrity in Alzheimer's Disease vs. Late Onset Bipolar Disorder and Its Correlation with Systemic Inflammation and Oxidative Stress Biomarkers. Frontiers in aging neuroscience. 2017;9:179.

13. Lesh TA, Careaga M, Rose DR, McAllister AK, Van de Water J, Carter CS, et al. Cytokine alterations in first-episode schizophrenia and bipolar disorder: relationships to brain structure and symptoms. Journal of neuroinflammation. 2018;15(1):165.

14. Poletti S, Leone G, Hoogenboezem TA, Ghiglino D, Vai B, de Wit H, et al. Markers of neuroinflammation influence measures of cortical thickness in bipolar depression. Psychiatry research Neuroimaging. 2019;285:64-6.

15. Tsai SY, Gildengers AG, Hsu JL, Chung KH, Chen PH, Huang YJ. Inflammation associated with volume reduction in the gray matter and hippocampus of older patients with bipolar disorder. Journal of affective disorders. 2019;244:60-6.

16. Furlan R, Melloni E, Finardi A, Vai B, Di Toro S, Aggio V, et al. Natural killer cells protect white matter integrity in bipolar disorder. Brain, behavior, and immunity. 2019;81:410-21.

17. King S, Jelen LA, Horne CM, Cleare A, Pariante CM, Young AH, et al. Inflammation, Glutamate, and Cognition in Bipolar Disorder Type II: A Proof of Concept Study. Frontiers in psychiatry. 2019;10:66.

18. Chen MH, Chang WC, Hsu JW, Huang KL, Tu PC, Su TP, et al. Correlation of proinflammatory cytokines levels and reduced gray matter volumes between patients with bipolar disorder and unipolar depression. Journal of affective disorders. 2019;245:8-15.

19. Chen P, Chen F, Chen G, Zhong S, Gong J, Zhong H, et al. Inflammation is associated with decreased functional connectivity of insula in unmedicated bipolar disorder. Brain, behavior, and immunity. 2020;89:615-22.

20. Mansur RB, Delgado-Peraza F, Subramaniapillai M, Lee Y, Iacobucci M, Rodrigues N, et al. Extracellular Vesicle Biomarkers Reveal Inhibition of Neuroinflammation by Infliximab in Association with Antidepressant Response in Adults with Bipolar Depression. Cells. 2020;9(4).

21. Poletti S, Mazza MG, Vai B, Lorenzi C, Colombo C, Benedetti F. Proinflammatory Cytokines Predict Brain Metabolite Concentrations in the Anterior Cingulate Cortex of Patients With Bipolar Disorder. Frontiers in psychiatry. 2020;11:590095.

22. Shonibare DO, Patel R, Islam AH, Metcalfe AWS, Fiksenbaum L, Kennedy JL, et al. Preliminary study of structural magnetic resonance imaging phenotypes related to genetic variation in Interleukin-1β rs16944 in adolescents with Bipolar Disorder. Journal of psychiatric research. 2020;122:33-41.

23. Bond DJ, Torres IJ, Lam RW, Yatham LN. Serum epidermal growth factor, clinical illness course, and limbic brain volumes in early-stage bipolar disorder. Journal of affective disorders. 2020;270:30-5.

24. Bai YM, Chen MH, Hsu JW, Huang KL, Tu PC, Chang WC, et al. A comparison study of metabolic profiles, immunity, and brain gray matter volumes between patients with bipolar disorder and depressive disorder. Journal of neuroinflammation. 2020;17(1):42.

25. Chen MH, Kao ZK, Chang WC, Tu PC, Hsu JW, Huang KL, et al. Increased Proinflammatory Cytokines, Executive Dysfunction, and Reduced Gray Matter Volumes In First-Episode Bipolar Disorder and Major Depressive Disorder. Journal of affective disorders. 2020;274:825-31.

26. Quidé Y, Bortolasci CC, Spolding B, Kidnapillai S, Watkeys OJ, Cohen-Woods S, et al. Systemic inflammation and grey matter volume in schizophrenia and bipolar disorder: Moderation by childhood trauma severity. Progress in neuro-psychopharmacology & biological psychiatry. 2021;105:110013.

27. Tang G, Chen P, Chen G, Zhong S, Gong J, Zhong H, et al. Inflammation is correlated with abnormal functional connectivity in unmedicated bipolar depression: an independent component analysis study of resting-state fMRI. Psychological medicine. 2021:1-11.

28. Mohite S, Salem H, Cordeiro T, Tannous J, Mwangi B, Selvaraj S, et al. Correlations between peripheral levels of inflammatory mediators and frontolimbic structures in bipolar disorder: an exploratory analysis. CNS spectrums. 2021:1-6.

29. Strenn N, Pålsson E, Liberg B, Landén M, Ekman A. Influence of genetic variations in IL1B on brain region volumes in bipolar patients and controls. Psychiatry research. 2021;296:113606.

30. Tseng HH, Chang HH, Wei SY, Lu TH, Hsieh YT, Yang YK, et al. Peripheral inflammation is associated with dysfunctional corticostriatal circuitry and executive dysfunction in bipolar disorder patients. Brain, behavior, and immunity. 2021;91:695-702.

31. Comai S, Melloni E, Lorenzi C, Bollettini I, Vai B, Zanardi R, et al. Selective association of cytokine levels and kynurenine/tryptophan ratio with alterations in white matter microstructure in bipolar but not in unipolar depression. European neuropsychopharmacology : the journal of the European College of Neuropsychopharmacology. 2022;55:96-109.

32. Gong J, Chen G, Chen F, Zhong S, Chen P, Zhong H, et al. Association between resting-state functional connectivity of amygdala subregions and peripheral pro-inflammation cytokines levels in bipolar disorder. Brain imaging and behavior. 2022;16(4):1614-26.

33. Jiang X, Guo Y, Jia L, Zhu Y, Sun Q, Kong L, et al. Altered Levels of Plasma Inflammatory Cytokines and White Matter Integrity in Bipolar Disorder Patients With Suicide Attempts. Frontiers in psychiatry. 2022;13:861881.

34. Bond DJ, Andreazza AC, Torres IJ, Honer WG, Lam RW, Yatham LN. Association of total peripheral inflammation with lower frontal and temporal lobe volumes in early-stage bipolar disorder: A proof-of-concept study. Journal of affective disorders. 2022;319:229-34.
